# Electrochemically Driven Phase Transition in LiCoO_2_ Cathode

**DOI:** 10.3390/ma14020242

**Published:** 2021-01-06

**Authors:** Jinhui Tan, Zhongzui Wang, Guangzhao Li, Huicong Hu, Jie Li, Rui Han, Dongyan Zhang

**Affiliations:** 1 School of Automobile and Transportation, Xihua University, Chengdu 610039, China; jinhuit@163.com; 2 School of Materials Science and Engineering, Xihua University, Chengdu 610039, China; wzz0901@163.com (Z.W.); guangzhao.li@hotmail.com (G.L.); 3 School of Advanced Materials and Nanotechnology, Xidian University, Xi’an 710126, China; huiconghu@stu.xidian.edu.cn (H.H.); lj521626@163.com (J.L.); 4 School of Optoelectronic Science and Engineering, University of Electronic Science and Technology of China, Chengdu 610054, China

**Keywords:** LiCoO_2_, degradation, Co_3_O_4_, phase transition

## Abstract

Lithium cobalt oxide (LiCoO_2_), which has been successfully applied in commercial lithium-ion batteries for portable devices, possesses a theoretical specific capacity of 274 mAh g^−1^. However, its actual capacity is only half of the theoretical specific capacity, because the charging voltage is restricted below 4.2 V. If a higher charging voltage is applied, an irreversible phase transition of LiCoO_2_ during delithiation would occur, resulting in severe capacity fading. Therefore, it is essential to investigate the electrochemically driven phase transition of LiCoO_2_ cathode material to approach its theoretical capacity. In this work, it was observed that LiCoO_2_ partially degraded to Co_3_O_4_ after 150 charging-discharging cycles. From the perspective of crystallography, the conventional cell of LiCoO_2_ was rebuilt to an orthonormal coordinate, and the transition path from layered LiCoO_2_ to cubic Co_3_O_4_ proposed. The theoretical analysis indicated that the electrochemically driven phase transition from LiCoO_2_ to Co_3_O_4_ underwent several stages. Based on this, an experimental verification was made by doping LiCoO_2_ with Al, In, Mg, and Zr, respectively. The doped samples theoretically predicted behavior. The findings in this study provide insights into the electrochemically driven phase transition in LiCoO_2_, and the phase transition can be eliminated to improve the capacity of LiCoO_2_ to its theoretical value.

## 1. Introduction

Lithium-ion batteries (LIBs) are widely implemented in mobile equipment and electric vehicles to provide power because of their high power density and cycle stability [1,2,3,4]. In general, cathode materials are the major factor for the performance of LIBs, and higher energy density and better cycle stability are pursued [5,6,7,8,9,10]. Layered lithium cobalt oxide (LiCoO_2_) is considered as one of the most important cathode materials due to its large theoretical capacity of 274 mAh g^−1^ [11,12]. LiCoO_2_ operates by reversible de-intercalation (charge) and intercalation (discharge) of lithium ions, without the irreversible phase transition of crystal structures [13,14,15]. The charging voltage of LiCoO_2_ cathode is restricted to 4.2 V to avoid the irreversible phase transition. Due to this limitation, commercial LiCoO_2_ only exhibits a little more than half of its theoretical value [1,16]. The irreversible phase transition arising from over charging voltage would cause the cathode capacity to fade quickly [17,18,19]. Therefore, to further improve the performance of LiCoO_2_, e.g., charging voltage, capacity, and cycling stability, great efforts should be made to inhibit the irreversible phase transition during charge/discharge cycles [20]_._ Although various beneficial modifications have been reported in recent years [21,22,23], there is a lack of insight into the origin of electrochemically driven phase transition in LiCoO_2_. Hence, currently, LiCoO_2_ is still unable to be charged to a higher voltage and its capacity is much less than its theoretical value.

Van der Ven et al [24] theoretically studied the phase stability in Li_x_CoO_2_ with different lithium contents and predicted that Li_x_CoO_2_ went through a phase transition from O3 to H1-3 symmetries as lithium content decreased. Chen et al [25] verified the phase transition in Li_x_CoO_2_ with decreasing lithium content by in-situ XRD. Dahéron et al [26] and Ohnishi et al [27] found the degraded product of CoxOy from LiCoO_2_ due to the irreversible electrochemically driven phase transition. However, the phase of Co_x_O_y_ is still controversial. Electrochemically driven phase transition path in LiCoO_2_ is still unclear. It is widely acknowledged that an effective strategy to improve the capacity of LiCoO_2_ is to inhibit its phase transition during delithiation. Liu et al [28] improved the capacity of LiCoO_2_ to 190 mAh g^−1^ with 96% capacity retention over 50 cycles by inhibiting the order-disorder transition and H1-3 transition. Therefore, it is of utmost significance to determine the mechanism of the electrochemically driven phase transition in LiCoO_2_ during charge/discharge cycles, to further improve the cycling stability and practical capacity of LiCoO_2_.

In this work, the electrochemically driven phase transition of LiCoO_2_ during charge/discharge cycles was investigated. The microstructure change of LiCoO_2_ after 150 cycles at charging voltage of 4.3 V was observed by high resolution transmission electron microscope (HRTEM). From the perspective of crystallography, the conventional cell of LiCoO_2_ was rebuilt to an orthonormal coordinate, the Co and O sites in LiCoO_2_ and Co_3_O_4_ were compared, and the electrochemically driven phase transition route was proposed. Both experimental observation and theoretical analysis indicated that the electrochemically driven phase transition occurred at the surface of LiCoO_2_ particle. Furthermore, relevant experiments were designed to verify the proposed transition mechanism.

## 2. Experimental Procedure and Computational Method

### 2.1. Samples Preparation

In this study, LiCoO_2_ powders were synthesized by sintering the oxide mixture of Li_2_CO_3_ and Co_3_O_4_ at 1000 °C for 12 h. Then, the as-prepared LiCoO_2_ was used as cathode in the Li-ion coin-cell, in which the counter electrode was metallic lithium foil, 1M LiPF_6_ was electrolyte, and Celgard 2300 was separator. After 150 charging-discharging cycles under 2.8–4.3 V at 0.5C, the cell was disassembled, and the electrode was removed. Then, the cathode powder was stripped and washed by alcohol for microstructure characterization by HRTEM. 

The doped LiCoO_2_ powders were synthesized by the same procedure, using Al_2_O_3_, In_2_O_3_, MgO, and ZrO_2_ as dopants. The doping content was controlled at 1 mol% in all samples. To characterize the electrochemical performance, the doped or undoped LiCoO_2_ powders were mixed with the solvent system including 10 wt% acetylene black, 10wt% PVDF and N-methyl-2-pyrrolidine (NMP) to prepare a homogenous slurry. The slurry was casted on Al foil, dried at 120 °C and employed as a cathode in 2032-type Li-ion coin-cell. The coin-cells were assembled by using Celgard 2300 as a separator, a counter electrode of metallic lithium foil, and LiPF_6_ dissolved in the mixed organic solvent of ethylene carbonate, dimethyl carbonate, and diethyl carbonate to obtain 1M solution as the electrolyte.

### 2.2. Characterization

The microstructure was observed by transmission electron microscopy (TEM, JEM 2100F, JEOL, Tokyo, Japan). X-ray photoelectron spectroscopy (XPS, Thermo Fisher ESCALAB Xi+, Waltham, MA, USA) analysis was performed to determine the elemental valence states. The crystalline phase of as-prepared powders was identified by X-ray diffraction (XRD, Bruker D8 Advanced, Hamburg, Germany) in standard θ–2θ configuration, using Cu Kα radiation. The galvanostatic charge-discharge performance was measured in the potential range of 2.0–4.3 V (vs. Li/Li^+^) at room temperature by using a battery test system (Land CT2001A, Wuhan Jinnuo Electronic Co. Ltd., Wuhan, China).

### 2.3. Density Functional Theory Calculations

A first-principles calculation was performed by using the Vienna ab initio simulation package (VASP) [29] within the density functional theory (DFT) using the supplied PAW pseudopotentials [30,31] and the Perdew–Burke–Ernzerhof generalized gradient approximation (PBE-GGA) [32]. Cut-off energy for the plane wave basis was chosen as 570 eV and 6 × 6 × 6 Monkhorst-Pack k grid was used for sampling the Brillouin zone. Structure optimization was performed with the convergence criteria that energy change was less than 1 × 10^−4^ eV before calculating the energy at each transition step.

## 3. Results and Discussion

### 3.1. Microstructure Characterization

**Figure 1:**
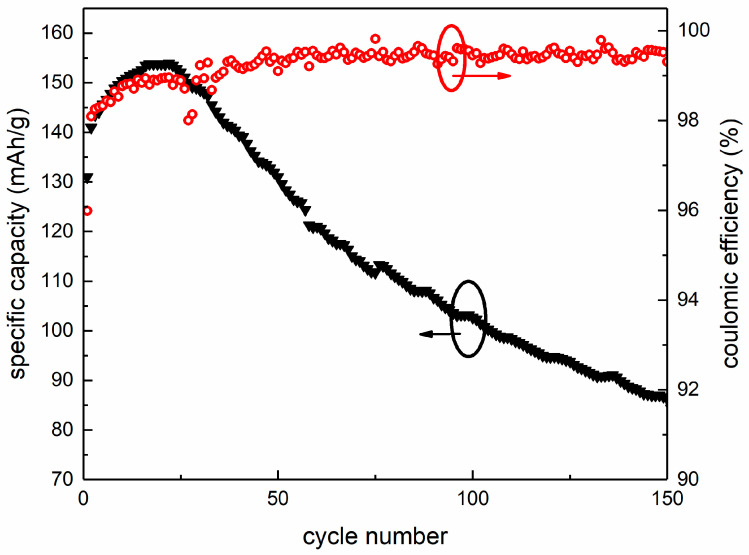
The Coulombic efficiency of Li-ion coin-cell with LiCoO_2_ as cathode materials.

**Figure 2:**
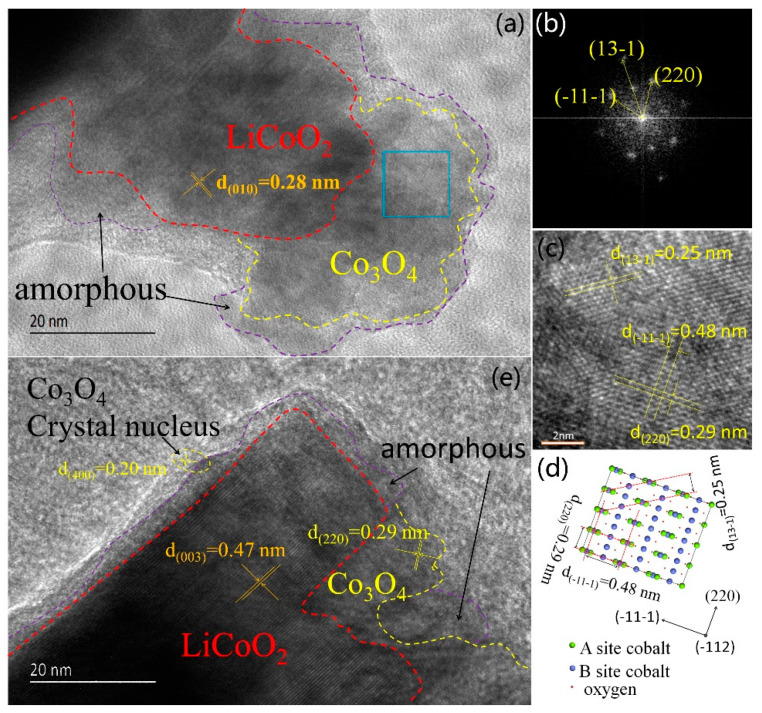
High resolution transmission electron microscope (HRTEM) images and structure analysis of LiCoO_2_ after 150 charging-discharging cycles under 4.3 V. (**a**) HRTEM image consists of LiCoO_2_, Co_3_O4 and amorphous region, (**b**) HRTEM image consist of LiCoO_2_, Co_3_O_4_, Co_3_O_4_ crystal nucleus and amorphous region, (**c**) FFT of the blue square selected region in (**a**); (**d**) inverse FFT of (**c**); (**e**) the atomic model of Co_3_O_4_ $\left(\bar{1}12\right)$ surface.

The as-prepared coin-cell was operated under 2.8–4.3 V (vs. Li/Li^+^) and was then disassembled to evaluate the degradation of LiCoO_2_ cathode. Figure 1 shows the Coulombic efficiency of the as-prepared Li-ion coin-cell with LiCoO_2_ as cathode. At the beginning, the Coulombic efficiency was low because the high charging voltage caused the phase transition of LiCoO_2_ cathode material. As a result, Li ions failed to re-intercalate into the cathode. In order to investigate the electrochemically driven phase transition of LiCoO_2_ cathode, the degradation of LiCoO_2_ cathode from disassembled cell was observed by HRTEM, and the obtained images are shown in Figure 2. Three different phase regions were observed on the LiCoO_2_ particle surface, namely LiCoO_2_ crystal, Co_3_O_4_ crystal and amorphous, respectively, as shown in Figure 2a,b. Also, Figure 2b shows a very small crystallite inclusion indicated by the yellow dash circle which is considered to be Co_3_O_4_ crystal nucleus. Figure 2c shows the corresponding fast Fourier transformation (FFT) of the square selected area in Figure 2a. Analysis of FFT pattern indicates that the phase constitution of the square selected area in Figure 2a is Co_3_O_4_ (PDF#80-1540) with Fd-3m symmetry. Figure 2d,e are inverse FFT of Figure 2b and atomic configuration of Co_3_O_4_ $\left(\bar{1}12\right)$ surface, respectively. Microstructure analysis of the degradation of LiCoO_2_ cathode revealed that a phase transition occurred from layered LiCoO_2_ to cubic Co_3_O_4_ in the surface zone of LiCoO_2_ cathode. 

**Figure 3:**
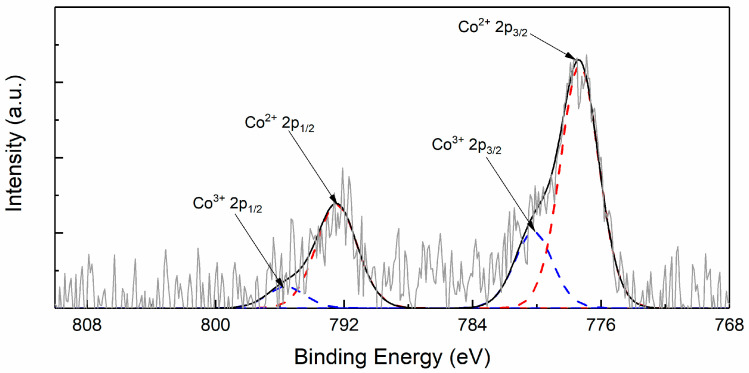
XPS of LiCoO_2_ after 150 charging-discharging cycles under 4.2 V.

XPS analysis was employed to determine the valence state of Co element during the degradation of LiCoO_2_ cathode, as shown in Figure 3. The surface of degraded LiCoO_2_ particles contained Co^2+^ and Co^3+^ ions, with more Co^2+^ than Co^3+^. In contrast, pristine LiCoO_2_ before charge-discharge cycling only contained Co^3+^ ions. Apparently, Co^2+^ ions were produced from the phase transition of LiCoO2 during the process of delithiation-lithiation, and originated from electrochemically driven phase transition product, Co_3_O_4_. This result further indicated the surface phase transition from layered LiCoO_2_ to cubic Co_3_O_4_. 

### 3.2. Modeling and Density Functional Theory Calculations: 

According to the TEM analysis, surface composition of as-prepared LiCoO_2_ particles degraded into Co_3_O_4_ after 150 cycles in the potential range of 2.8~4.3 V. During the charge process, de-intercalation of Li-ions occurred as follows:

(1) $${\mathrm{LiCoO}}_{2}\;\begin{array}{c} \overset{\;\mathrm{charging}\;}{\to }\\ \underset{\mathrm{discharging}}{\leftarrow } \end{array}{\;\mathrm{xLi}}^{+}+{\mathrm{Li}}_{1-x}{\left({\mathrm{CoO}}_{2}\right)}^{-}$$

During the discharge process, an inverse reaction of Equation (1) occurred. However, some Li-ions failed to be intercalated into the layered structure of cathode, resulting in capacity fading, as shown in Figure 2. The failure of Li-ions to re-intercalate was attributed to the structural transformation of layered (CoO_2_)^−^ during delithiation, which is an irreversible phase transition causing degradation.

(2) $${\mathrm{LiCoO}}_{2}\overset{\mathrm{charging}(\mathrm{high}\;\mathrm{voltage})}{\to }{\mathrm{Li}}^{+}+\frac{1}{3}O_{2}↑+\frac{1}{3}{\mathrm{Co}}_{3}O_{4}$$

**Figure 4:**
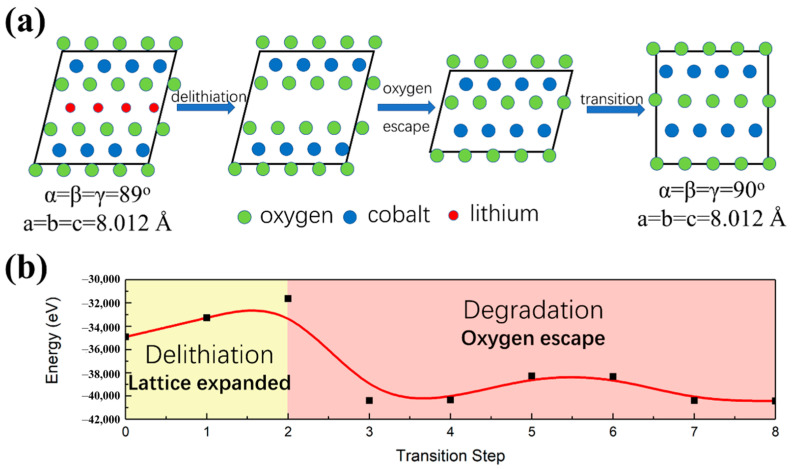
(**a**) Schematic diagram of electrochemically driven phase transition path from LiCoO_2_ to Co_3_O_4_; (**b**) calculated potential barrier along the proposed electrochemically driven phase transition path from LiCoO_2_ to Co_3_O_4_.

Comparing the structure of layered (CoO_2_)^−^ lacking lithium ion with that of Co_3_O_4_, it was inferred that the following two steps likely occurred: (1) Li-ions were released from cathode under external field, and the lattice of (CoO_2_)^−^ expanded; (2) lattice oxygen escaped from the layered (CoO_2_)^−^, forming amorphous Co_x_O and then transforming to Co_3_O_4_. Figure 4 schematically shows the de-intercalation of Li-ions from LiCoO_2_ and the electrochemically driven phase transition from LiCoO_2_ to Co_3_O_4_, DFT calculation was performed according to the proposed transition path. In order to provide insights into the mechanism of degradation of LiCoO_2_ to Co_3_O_4_, the crystal cell of layered LiCoO_2_ was rebuilt from a hexagonal presentation to an orthogonal coordination, as shown in Figure 4a. After comparing each atom position in (CoO_2_)^−^ and Co_3_O_4_, a first principles calculation was performed based on DFT [33,34]. In the rebuilt LiCoO_2_ cell, 64 atoms were employed to perform the DFT calculation. The occupancy and displacement of atoms in rebuilt LiCoO_2_ cell were gradually changed to approach Co_3_O_4_ structure in order to simulate the transition path and to calculate the potential barrier during transition. Figure 4b shows the calculated total energy along the transition path from LiCoO_2_ to Co_3_O_4_. Under an external electric field, LiCoO_2_ overcame the potential barrier at transition steps 1 and 2 to release Li-ions. Accompanying the release of Li-ions, partial loss of lattice oxygen and lattice expansion occurred. The oxygen escaped from lattice because the applied voltage made the Fermi level touch O 2p level. Due to the existence of the potential barrier, this reaction was driven by an external electric field.

**Figure 5:**
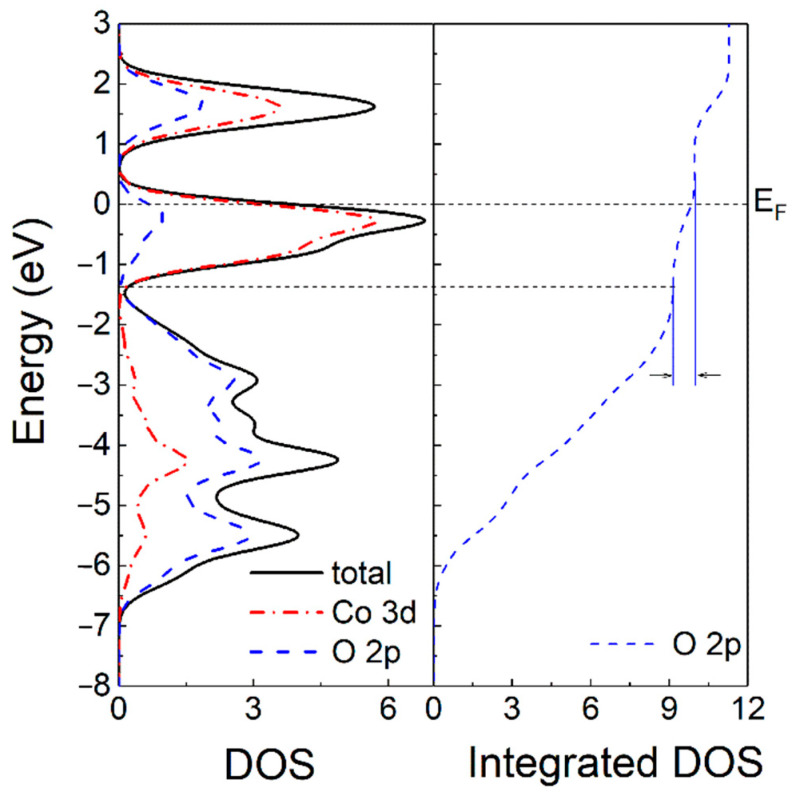
Calculated density of states (DOS) and integrated DOS of LiCoO_2_.

Figure 5 shows the calculated density of states (DOS) and integrated DOS of LiCoO_2_. First principles calculation indicates that LiCoO_2_ is a compound with significant covalent character [35]. The interaction of Co 3d-electrons with O 2p-electrons resulted in a splitting of the 3d levels and a hybridization of Co 3d and O 2p levels. Under an external field, Li-ions deintercalated from layered LiCoO_2_ cathode, which lowered the Fermi level to touch O 2p level. This led to the generation of holes in the bonding anion p-state. Thus, oxidation reaction of anions occurred, causing the decomposition of (CoO_2_)^−^ by oxygen loss. In LiCoO_2_ cathode, oxygen atoms form an interstitial octahedron, and cobalt atoms occupy the center. Li-ions take positive electricity, and Co-O octahedrons take negative electricity. When Li-ions were released from LiCoO_2_ cathode, the lattice expanded due to the electrostatic repulsion between Co-O octahedrons. Hence, partial oxygen loss and lattice expansion led to degradation of LiCoO_2_ into amorphous Co_x_O_y_ which was observed in Figure 2e. Besides, CoxO eventually formed Co_3_O_4_ which contributed to the capacity fading. As can be seen, in the integrated DOS for O atoms, the hybridization part of O-2p orbitals with Co-3d orbitals above −1.5 eV was less than one electron per unit cell. This indicated that an appropriate applied voltage would not lead to oxygen loss. The experimental results also confirmed that LiCoO_2_ cathode exhibited good cycling stability when the charge voltage was at 4.2 eV. The calculated energy barrier along transition path indicated that the degradation process should overcome a potential barrier to finally form Co_3_O_4_. Consequently, in order to maintain the layered structure of LiCoO_2_ and to improve its charge voltage and capacity, its lattice should be compressed by doping ions with smaller radius and the hole capture level should be introduced by doping ions with lower valence, which was confirmed by our previous work [36].

### 3.3. Experimental Verification

From the aforementioned microstructure analysis and first principle calculation, it can be inferred that two key issues prompt the irreversible transition of LiCoO_2_ after releasing lithium. One is lattice expansion, and the other is O^2−^ oxidation. If these two factors are inhibited, the cycling stability of LiCoO_2_ cathode material will be improved. Doping ions with smaller radius would inhibit the lattice expansion, while doping ions with lower valence would trap more holes. Therefore, through doping, the performance of LIBs would be improved or deteriorated at charge voltage above 4.2 V. To verify the theoretical analysis, LiCoO_2_ was doped with 1 mol% of Al, In, Mg, and Zr, respectively.

**Figure 6:**
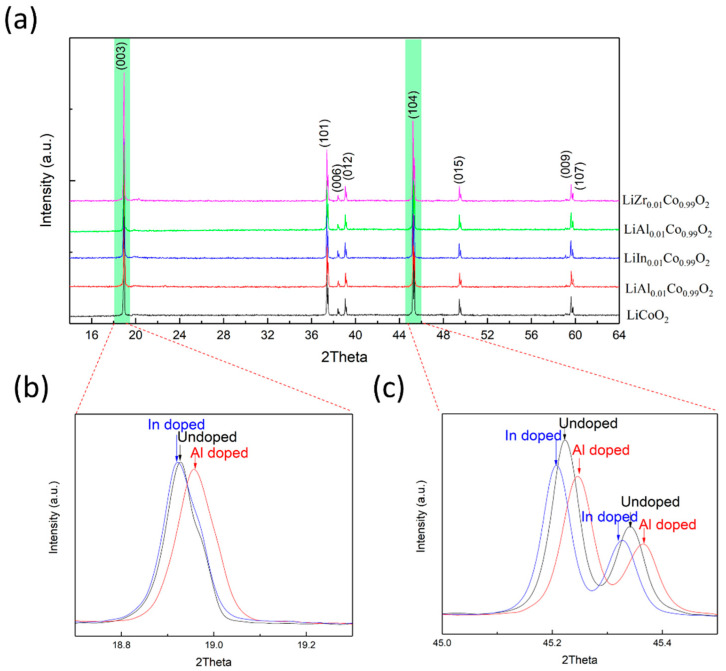
(**a**) XRD patterns of doped and undoped LiCoO_2_. (**b**) Enlarged view of (003) peak. (**c**) Enlarged view of (104) peak.

The experimental result based on the theoretical prediction are listed in Table 1. Among the doping ions, Al and In ions have the same valence state of +3 as Co ions in LiCoO_2_, but the radius of Al^3+^ ions is smaller than that of Co^3+^ and the radius of In^3+^ ions is larger than that of Co^3+^. Hence, Al doping can compress the LiCoO_2_ lattice and inhibit the phase transition. In doping will expand the LiCoO_2_ lattice, promoting the phase transition which would cause performance deterioration. Mg^2+^ and Zr^4+^ have similar ionic radius compared to Co^3+^, but different valence state. Mg doping will introduce the acceptor level which will enable trapping of holes, and thus inhibit O^2−^ oxidation. Zr doping will form donor level due to higher valence, which will compensate the intrinsic acceptor level to promote the O^2−^ oxidation [36]. Figure 6 shows the XRD patterns of doped and undoped LiCoO_2_ samples. As can be seen, doping with 1% Al, In, Mg and Zr did not change the phase of LiCoO_2_. The inset is the enlarged view of (003) and (104) peaks of Al and In doped samples. It can be seen that both the (003) and (104) peaks of Al doped sample shifted towards higher angles, which indicated that the lattice shrunk according to the Bragg equation, while the In doped sample showed the effect of lattice expansion. The calculated lattice parameters and their variation are shown in Table 2.

**Figure 7:**
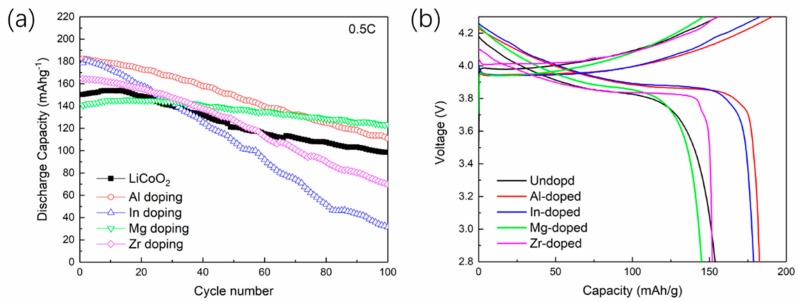
(**a**) Cycling performance comparison; (**b**) The charge-discharge profiles of doped and undoped LiCoO_2_.

Figure 7 shows the cycling performance and charge-discharge profiles of doped and undoped LiCoO_2_ samples. The galvanostatic charge-discharge performance was measured at 0.5 C by charging to 4.3 V. After 100 cycles, Al and Mg doped LiCoO_2_ exhibited better capacities, while In and Zr doped LiCoO_2_ exhibited worse capacities, in agreement with the theoretical prediction. This verifies the theoretical analysis on the electrochemically driven phase transition from LiCoO_2_ to Co_3_O_4_. Notably, Al doped LiCoO_2_ demonstrated good cycling stability under 0.5 C after 100 cycles, and a specific capacity as high as 180 mAh g^−1^ during the initial cycles. Therefore, the capacity and cycling stability can be effectively improved through our proposed mechanism. Namely, the electrochemically driven phase transition can be inhibited by doping elements with smaller radius or lower valence.

## 4. Conclusions

In this study, HRTEM observations of LiCoO_2_ cathode particle were performed after 150 charging-discharging cycles under 2.8–4.3 V. It was found that the surface of LiCoO_2_ particle consisted of LiCoO_2_ crystal, Co_3_O_4_ crystal, Co_3_O_4_ crystal nucleus, and an amorphous region. According to the degradation product Co_3_O_4_ from LiCoO_2_, DFT calculations were performed to investigate the electrochemically driven phase transition path. Theoretical analysis indicated that the electrochemically driven phase transition undergoes two stages, lattice expansion, and oxygen escape. Through doping, the electrochemically driven phase transition of LiCoO_2_ could be inhibited and the electrochemical performance could be improved. The experimental verification was made by doping LiCoO_2_ with Al, In, Mg, and Zr to confirm the proposed electrochemically driven phase transition mechanism. Al or Mg doping significantly improved the cycling stability of LiCoO_2_ cathode materials by inhibiting the lattice expansion or oxygen escape, respectively. 

## Figures and Tables

**Table 1:** The theoretical prediction, experimental results, and literature results of the cycling performance for doped LiCoO_2_.

Doping Ion	Ion Radius (Å)	Effect	Theoretical Prediction	Experimental Results	Literature Results
Al^3+^	0.50	Inhibit lattice expansion	Improved	Better	Better [37,38]
In^3+^	0.81	Promote lattice expansion	Deteriorated	Worse	-
Mg^2+^	0.72	Inhibit oxidation	Improved	Better	Better [20]
Zr^4+^	0.72	Promote oxidation	Deteriorated	Worse	Worse [39]
Co^3+^ (Reference)	0.745	Reference			

**Table 2:** Calculated lattice parameters of doped LiCoO_2_, and their variation compared to the undoped LiCoO_2_.

Doped Element	Lattice a (Å)	Δa	Lattice c	Δc (Å)
Al	2.8147(4)	−0.051%	14.0458(5)	−0.042%
In	2.8169(7)	0.028%	14.0569(3)	0.037%
Mg	2.8160(4)	−0.005%	14.0535(9)	0.013%
Zr	2.8159(6)	−0.005%	14.0493(5)	−0.018%
undoped (Reference)	2.8161(8)	-	14.0517(8)	-

## Data Availability

The data presented in this study are available on request from the corresponding author.
